# Comparative Transcriptome Analysis Combining SMRT- and Illumina-Based RNA-Seq Identifies Potential Candidate Genes Involved in Betalain Biosynthesis in Pitaya Fruit

**DOI:** 10.3390/ijms21093288

**Published:** 2020-05-06

**Authors:** Yawei Wu, Juan Xu, Xiumei Han, Guang Qiao, Kun Yang, Zhuang Wen, Xiaopeng Wen

**Affiliations:** 1 Key Laboratory of Plant Resource Conservation and Germplasm Innovation in Mountainous Region (Ministry of Education), Institute of Agro-Bioengineering/College of Life Sciences, Guizhou University, Guiyang 550025, Guizhou, China; yaweiwu2006@163.com (Y.W.); gqiao@gzu.edu.cn (G.Q.); kyanggz@163.com (K.Y.); gzu_zwen@163.com (Z.W.); 2 Institute of Pomology Science, Guizhou Academy of Agricultural Sciences, Guiyang 550006, Guizhou, China; xiaocao550100@163.com; 3 Key Laboratory of Horticultural Plant Biology (Ministry of Education), Huazhong Agricultural University, Wuhan 430070, Hubei, China; xujuan@mail.hzau.edu.cn

**Keywords:** pitaya, SMRT, betalain, different expressed genes, qRT-PCR

## Abstract

To gain more valuable genomic information about betalain biosynthesis, the full-length transcriptome of pitaya pulp from ‘Zihonglong’ (red pulp) and ‘Jinghonglong’ (white pulp) in four fruit developmental stages was analyzed using Single-Molecule Real-Time (SMRT) sequencing corrected by Illumina RNA-sequence (Illumina RNA-Seq). A total of 65,317 and 91,638 genes were identified in ‘Zihonglong’ and ‘Jinghonglong’, respectively. A total of 11,377 and 15,551 genes with more than two isoforms were investigated from ‘Zihonglong’ and ‘Jinghonglong’, respectively. In total, 156,955 genes were acquired after elimination of redundancy, of which, 120,604 genes (79.63%) were annotated, and 30,875 (20.37%) sequences without hits to reference database were probably novel genes in pitaya. A total of 31,169 and 53,024 simple sequence repeats (SSRs) were uncovered from the genes of ‘Zihonglong’ and ‘Jinghonglong’, and 11,650 long non-coding RNAs (lncRNAs) in ‘Zihonglong’ and 11,113 lncRNAs in ‘Jinghonglong’ were obtained herein. qRT-PCR was conducted on ten candidate genes, the expression level of six novel genes were consistent with the Fragments Per Kilobase of transcript per Million mapped reads (FPKM) values. In conclusion, we firstly undertook SMRT sequencing of the full-length transcriptome of pitaya, and the valuable resource that was acquired through this sequencing facilitated the identification of additional betalain-related genes. Notably, a list of novel putative genes related to the synthesis of betalain in pitaya fruits was assembled. This may provide new insights into betalain synthesis in pitaya.

## 1. Introduction

Pitaya (*Hylocereus*), originating from Latin America and the West Indies [1], is an economical and nutritional fruit cultivated in tropical and subtropical regions. *H. polyrhizus* (with red pulp and peel) and *H. undatus* (with white pulp and red peel) are the two major types [2]. Betacyanin and betaxanthin are the two major pigments of betalain, which mainly define the coloration in pitaya fruits [3]. Betalain is a tyrosine-derived pigment that occurs solely in the order of Caryophyllales, which largely replaces the anthocyanins in a mutually exclusive manner [4]. Betalain has high nutritional value and positive effects in health and disease prevention for high antioxidant and anti-inflammatory capabilities [5,6]. Therefore, betalain synthesis has become a research area of high scientific interest, as well as economic significance [7]. Currently, the metabolic pathway of betalain is clearly defined [4]. The initial step in betalain biosynthesis is the hydroxylation of tyrosine to form L-DOPA through the monophenolase activity of the enzyme tyrosinase, CYP76AD6, CYP76AD5, and the previously described CYP76AD1 [7,8]. However, CYP76AD1 is also able to produce cyclo-DOPA, CYP76AD5 and CYP76AD6 do not have this activity [9]. L-DOPA is subsequently converted to dopaquinone by CYP76AD1 [8,10], or it is alternatively converted to 4,5-seco-DOPA initiated by DOPA 4,5-dioxygenase (DOD) [11,12], and then, dopaquinone spontaneously converts to cyclo-DOPA and 4,5-seco-DOPA to betalamic acid identified as chromophore [6,13]. Next, betalamic acid condensates spontaneously with amino acid or amine to form betaxanthins, or with cyclo-DOPA to form betanidine, and betanidin is further glucosylated by a betanidin glucosyltransferase to form the basic betacyanins betanin or gomphrenin [4]. An alternative pathway was found, in which cyclo-DOPA is first glucosylated by a cyclo-DOPA-5-O-glucosyltransferase to form cDOPA-5-O-glucoside, and then condensates with betalamic acid to form betanin, which spontaneously condensates with betalamic acid for direct formation of betanin [4,14]. The expression of the key *BvDODA* and *BvCYP76AD1* are controlled by *BvMYB1* [15]. Expression of CYP76AD1, in combination with *BvDODA1* and cDOPA5GT, was therefore found to be sufficient for biosynthesis of betanin, without the need for an exogenous supply of L-DOPA [16]. Nevertheless, betalain biosynthesis has remained poorly understood in comparison to the other major classes of plant pigments. Then, the identification of new genes involved in betalain biosynthesis is important. Sequencing platforms is an efficient approach to identify putative genes; 9 key transcripts involved in betalain synthesis were identified based on Illumina RNA-Seq in pitaya [6]. Nonetheless, many questions, including betalain biosynthesis in pitaya remain open for limited sequence data [4].

Illumina RNA-Seq is a powerful tool for the description of gene expression levels [17]; however, it is difficult to identify full-length transcript using the Illumina RNA-Seq data [18]. High quality transcript sequences are crucial for plant biology research. Fortunately, full-length transcriptome is being employed as an effective approach to obtain high quality transcript sequences. Single-Molecule Real-Time (SMRT) sequencing developed by PacBio can obtain full-length sequencing without post-sequencing assembly [19,20], which has been used for whole-transcriptome profiling in many plants [21,22,23,24,25,26,27], but, thus far, not in pitaya. However, SMRT sequencing needs to be corrected with Illumina RNA-Seq reads to eliminate its high error rate [28]; hence, a combination of SMRT sequencing and Illumina RNA-Seq is a preferable process.

In the present work, a high quality full-length transcriptome of pitaya fruit was generated by combination of SMRT and Illumina RNA-Seq sequencing, and the transcript functional annotation, simple sequence repeats (SSRs) analysis and long non-coding RNAs (lncRNAs) prediction were performed based on the data. Putative genes involved in the biosynthesis of betalain were identified according to the characteristic of color phenotypic. This study might be a valuable resource for further investigation of pitaya, and might provide a better understanding of betalain biosynthesis in pitaya fruit.

## 2. Result

### 2.1. The Variation of Pulp Color Parameters and Betalain Content

**Figure 1:**
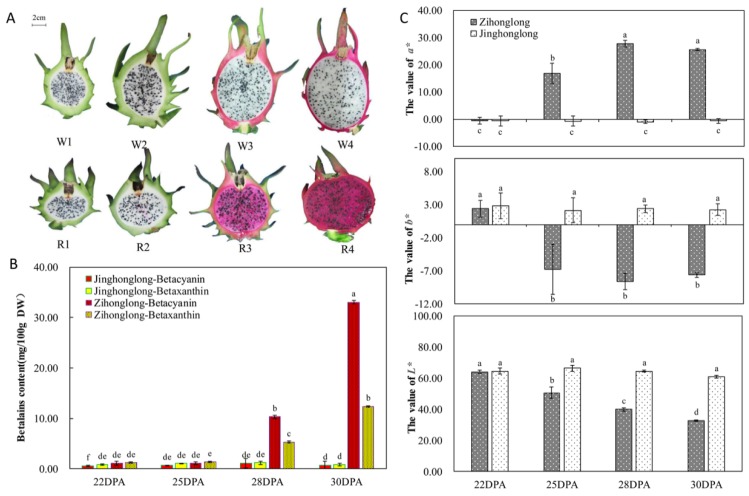
The variation of parameters related to pigmentation (**A**), Pulp color in four fruit development stages. (**B**), the content of betalain in pitaya fruit. (**C**), Color parameters of pitaya fruit. Note, in Figure 1A, W1 refers to ‘Jinghonglong’ fruit at 22 days post-anthesis (DPA) (stage 1). W2 refers to ‘Jinghonglong’ fruit at 25 DPA (stage 2). W3 refers to ‘Jinghonglong’ fruit at 28 DPA (stage 3). W4 refers to ‘Jinghonglong’ fruit at 30 DPA (stage 4). R1 refers to ‘Zihonglong’ fruit at 22 DPA (stage 1). R2 refers to ‘Zihonglong’ fruit at 25 DPA (stage 2). R3 refers to ‘Zihonglong’ fruit at 28 DPA (stage 3). R4 refers to ‘Zihonglong’ fruit at 30 DPA (stage 4), the same as follow. Different letters in Figure 1B and Figure 1C represent statistically significant differences (*p* < 0.05).

As shown in Figure 1A, cultivar apparently affected pulp color. As illustrated in Figure 1B, the concentration of betacyanin and betaxanthin in pulp of ‘Jinghonglong’ were very low and hardly varied during the four developmental stages; the former ranged from 0.56 (at 22 days post-anthesis (DPA)) to 1.09 mg/100 g DW (at 28 DPA) and the latter ranged from 0.82 mg/100 g DW (at 30 DPA) to 1.21 mg/100 g DW (at 28 DPA). From 22 to 25 DPA, a similar concentration and variation were found in ‘Zihonglong’; the concentration of betacyanin were both 1.08 mg/100 g DW and that of betaxanthin was varied from 1.18 to 1.38 mg/100 g DW. Furthermore, there were no significant differences between the two varieties in the concentration of betacyanin and betaxanthin of pulp at the two stages. In contrast, the concentration of betacyanin and betaxanthin in the pulp of ‘Zihonglong’ increased dramatically at 28 DPA (10.32 mg/100 g DW of betacyanin and 5.32 mg/100 g DW of betaxanthin, respectively), and reached peak values at 30 DPA (33.06 mg/100 g DW of betacyanin and 12.40 mg/100 g DW of betaxanthin, respectively). Moreover, the concentration of betacyanin was significantly higher than that of betaxanthin, which caused the red appearance of the pulp. There were no significant differences in the L*, a*, b* values of ‘Zihonglong’ and ‘Jinghonglong’ pulps at 22 DPA. The L*, a*, b* values of ‘Jinghonglong’ were relatively stable, while those of ‘Zihonglong’ changed remarkably during the fruit development stages. With the development of ‘Zihonglong’ fruit, L* value was decreased gradually, a* value increased prominently at 25 DPA, and reached the highest level, 27.90 at 28 DPA. There then appeared to be a slight decline for mature fruit. The b* value decreased from 2.11(yellow pulp) at 22 DPA to -6.73 (blue pulp) at 25 DPA (Figure 1C).

### 2.2. Transcriptome Analysis Using PacBio Sequel

The full-length transcriptome of pitaya fruit was generated by PacBio Sequel on ‘Zihonglong’ and ‘Jinghonglong’ (Table S1). In total, 9,579,839 subreads from 8.47 G bases were obtained from the pulp of ‘Zihonglong’, while 7,245,659 subreads were obtained from 7.74 G bases from the pulp of ‘Jinghonglong’. After removing adapters and artefacts, 367,001 circular consensus sequence (CCS) (including 314,173 full-length non-chimerics, FLNCs) of ‘Zihonglong’ and 481,602 CCS (including 348,184 FLNCs) of ‘Jinghonglong’ were generated, respectively. The lengths of ‘Zihonglong’ FLNCs ranged from 334 to 14,604 nt with an average length of 950 nt, while ‘Jinghonglong’ FLNCs showed an average length of 1095 nt and ranged from 374 to 6988 nt. For ‘Zihonglong’, 184,875 polished consensus sequences transcripts were produced, including 23,669 polished high-quality (HQ) and 161,206 low-quality (LQ) isoform sequences. For ‘Jinghonglong’, 188,215 polished consensus sequences, including 25,299 polished HQ and 162,916 LQ isoform sequences were obtained. After correcting and removing redundant reads, 65,312 and 91,638 genes (non-redundant reads) were obtained from full length transcripts of ‘Zihonglong’ and ‘Jinghonglong’, respectively.

### 2.3. Comparison of SMRT Sequencing and Next-Generation Sequencing

The number of SMRT gene obtained from SMRT sequencing were less than that of unigene assembled from Illumina RNA-Seq reads, whereas, the mean length of SMRT gene reached up to 1175 and 1337 nt in ‘Zihonglong’ and ‘Jinghonglong’, respectively, which are much longer than that of unigene assembled from Illumina RNA-Seq reads (681 nt in ‘Zihonglong’ and 696 nt in ‘Jinghonglong’). Regarding the assembled transcripts from RNA-seq reads, the percentage of bases <500, is about 65% in ‘Zihonglong’ and 74% in ‘Jinghonglong’. However, regarding the transcripts from the PacBio Sequel reads, the percentage of bases <500 is 12% in ‘Zihonglong’ and 6% in ‘Jinghonglong’. Approximately 80% of the transcripts from the PacBio Sequel reads ranged from 500 to 2000 bases (Table S2). Hence, the SMRT sequencing offered significant advantages over Illumina RNA-Seq in the length of reads, which provided more valuable transcripts data for the identification of putative genes involved in betalain biosynthesis in pitaya fruit.

### 2.4. Clustering Analysis

Multiple transcripts corresponded to one gene in the transcriptional group. PacBio long reads clustering analysis demonstrated that 65,317 and 91,638 genes were generated from polished consensus sequences transcripts in ‘Zihonglong’ and ‘Jinghonglong’, respectively. Various isoforms generated by a single gene were widely found among the tested samples. A total of 17.42% genes had more than one isoform in ‘Zihonglong’ pulp, which is slightly higher than that (16.97%) of ‘Jinghonglong’. In the former, 11,377 genes showed more than two alternative splice forms (isoforms), of which the majority corresponded to two-to-three isoforms, accounting for 74.78% of the total, and 516 genes contained over 10 isoforms. In the latter, 15,551 genes had more than two isoforms, among which the majority were two-to-three isoforms, accounting for 67.99% of the total, and 767 genes with over 10 isoforms were obtained (Figure S1). Therefore, when alleles and associated homologs were grouped against these results, they typically shared the same alternative splicing patterns [17], indicating that a gene might generate different transcripts via alternative splicing.

### 2.5. Function Annotation

Function annotation of pitaya non-redundant FLNC transcripts (genes) was investigated using different databases. As shown in Table 1, a total of 34,601 transcripts were annotated in the Clusters of Orthologous Groups of proteins (COG) database; 54,706 in Gene Ontology (GO); 28,796 in Kyoto Encyclopedia of Genes and Genomes (KEGG); 56,010 in euKaryotic Ortholog Groups (KOG); 88,549 in protein families and domains (Pfam); 72,130 in Swiss-Prot; 95,458 in TrEMBL; 10,5413 in Non-Redundant Protein Sequence Database (NR); and 63,052 in NCBI nucleic acid database (NT). Moreover, 120,604 transcripts were annotated in all of the nine databases, while 30,875 sequences without hits to reference database were probably novel genes in pitaya.

**Figure 2:**
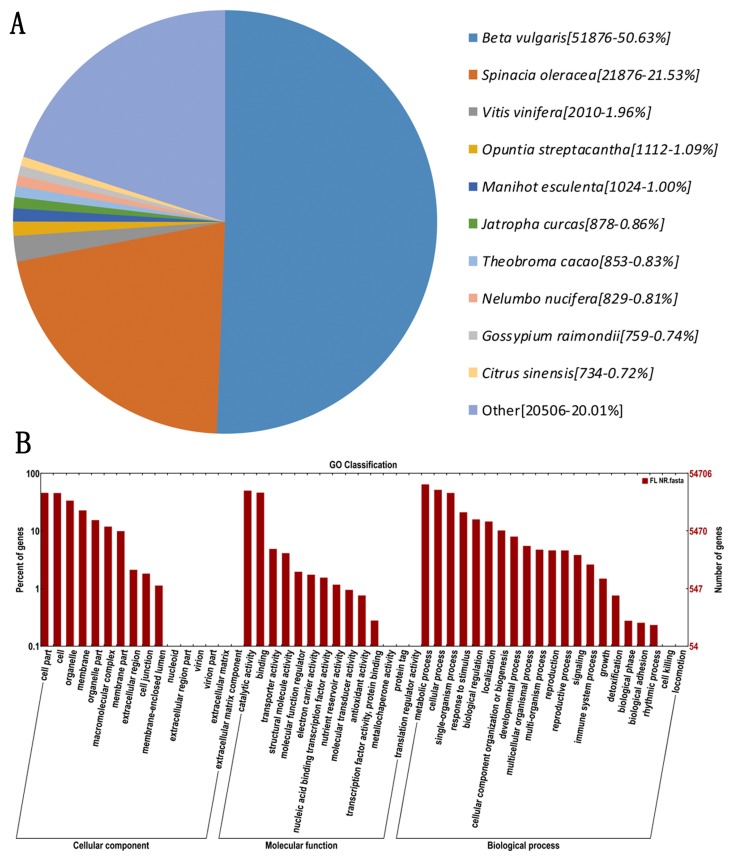
The distribution features of annotated genes. (**A**), Homologous species distribution of pitaya annotated in the NR database. (**B**), Gene Ontology (GO) functional annotation of pitaya genes. The x-axis represents GO categories, the y-axis (right) represents the number of genes, and the y-axis (left) represents the percentage of genes.

The homologous species of *Hylocereus* were predicted by sequence alignment on the basis of the NR database. Of all the genes hits to NR plant proteins from BLASTx, the pitaya genes gave the highest number of hits to the *Beta vulgaris* (51,879 hits), followed by *Spinacia oleraces* (21,876 hits), and *Vitis vinifere* (2010 hits) (Figure 2A). Most hits found in *Beta vulgaris* were probably due to pitaya and *Beta vulgaris* belonging to Caryophyllales, and the *Beta vulgaris* database being better annotated than those of other species. As shown in Figure 2B, the molecular function (MF, 62,439 FLNCs) was more abundant than biological process (BP, 142,635 FLNCs) and cellular component (CC, 104,215 FLNCs). Within these functional groups, the highest number of sequences were annotated with the metabolic process (35,263 sequences, 11.40%), cellular process (28,379 sequences, 9.18%), and catalytic activity (27,507 sequences, 8.89%). A total of 117 pathways with 28,796 genes were annotated by KEGG, associated with 23.88% of the whole annotated dataset (120,640 genes). Among these, 237 genes were identified in phenylalanine, tyrosine, and tryptophan biosynthesis pathway; however, a KEGG pathway involved in betalain biosynthesis was not found.

### 2.6. SSR and lncRNA Prediction

**Figure 3:**
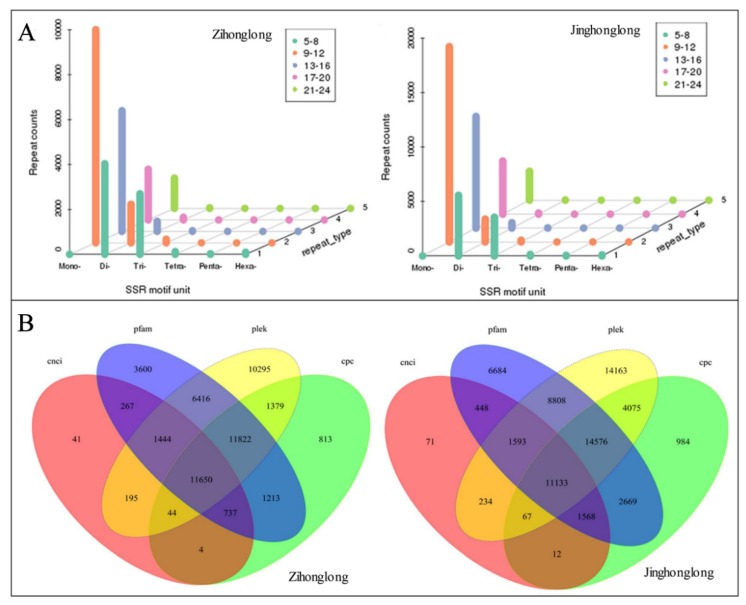
SSRs and lncRNAs of pitaya pulp. (**A**), The distribution characteristics of SSRs motifs. (**B**), Venn diagram of the number of lncRNAs predicted by Coding-Non-Coding Index (CNCI), pfam, Plek, and Coding Potential Calculator (CPC). Note, the number in Figure 3B represents the amount of lncRNAs.

A total of 31,169 SSRs were identified in 24,889 genes (38.10%) from ‘Zihonglong’, of which 11,885 genes contained more than one SSR, and the number of SSRs present in compound formation was 4472. A total of 53,024 SSRs were identified in 39,793 genes (43.42%) from ‘Jinghonglong’, of which 18,725 genes contained more than one SSR, and the number of SSRs present in compound formation was 8868 (Figure 3A). In both cases, the most abundant motifs detected was mono–nucleotides, accounting for 41.72% and 40.58% of the total SSRs in ‘Zihonglong’ and ‘Jinghonglong’, respectively, and 4883 (15.67%) and 6204 (11.70%) di-nucleotides were detected from ‘Zihonglong’ and ‘Jinghonglong’, respectively. We obtained 11,650 and 11,113 lncRNAs from 65,317 and 91,638 genes in ‘Zihonglong’ and ‘Jinghonglong’, respectively (Figure 3B). Four of these lncRNAs were up to 3000 nt in ‘Zihonglong’, while 18 up to 3000 nt were investigated from ‘Jinghonglong’, most of which were single-isoform transcripts presenting in both samples. The functions of these lncRNAs need to be further characterized.

### 2.7. Genes Involved in Betalain Biosynthesis

**Figure 4:**
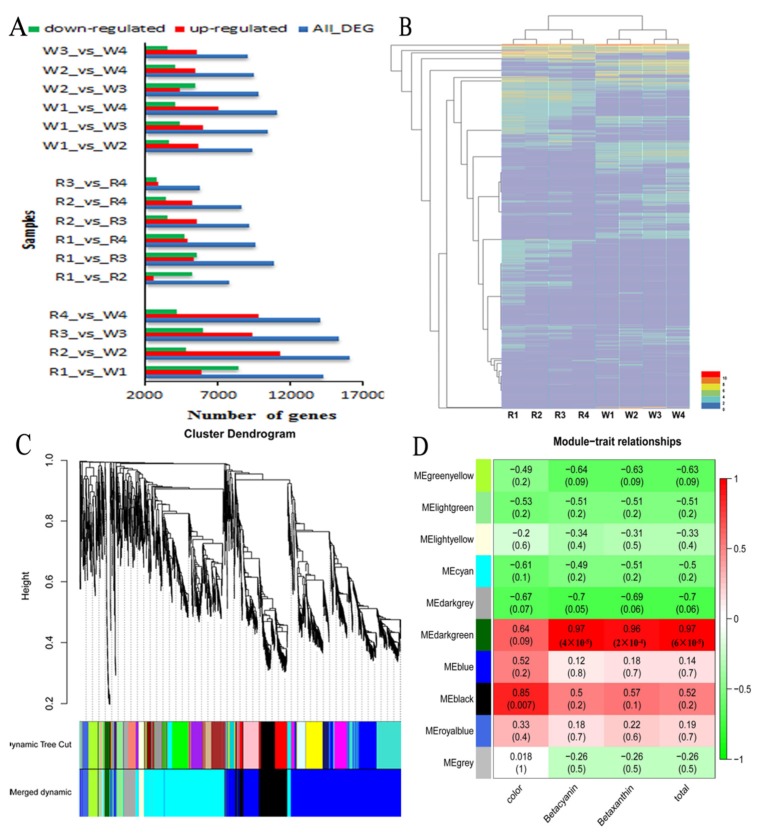
The identification of DEGs (**A**), Map of the number of differentially expressed genes; (**B**), Cluster map of differentially expressed genes; (**C**), Visualizing the gene network using a heatmap plot. The heatmap depicts the topological overlap matrix (TOM) among all genes in the analysis. (**D**), Module-trait associations. Each row corresponds to a module characteristic gene (eigengene), and each column corresponds to a trait.

Taking into account the different expression levels of genes between ‘Zihonglong’ and ‘Jinghonglong’, the genes from PacBio sequel were used as the reference dataset. It was shown that 44,109 differentially expressed genes (DEGs) were found between ‘Zihonglong’ and ‘Jinghonglong’ during four development stages, among which, most DEGs were investigated in R2_vs_W2, containing 11,317 up-regulated and 4788 down-regulated DEGs respectively (Figure 4A). The heat map of all DEGs in both ‘Zihonglong’ and ‘Jinghonglong’ was created, and the four developmental stages of ‘Zihonglong’ and ‘Jinghonglong’ were clustered, in both cultivars. The stage of 22 DPA and the 25 DPA were grouped together, and the stage of the 28 DPA and the 30 DPA were grouped together (Figure 4B). A total of 13,794 non-redundant DEGs with more than one Fragments Per Kilobase of transcript per Million mapped reads (FPKM) value above 10 were used to evaluate the candidate genes involved in betalain biosynthesis, and 10 modules were formed through weighted gene co-expression network analysis (WGCNA) (Figure 4C–E). Analysis of module-trait relationships revealed that the dark green and black modules containing 173 genes (Table S3) were highly correlated with the phenotypic traits of pitaya fruit.

**Figure 5:**
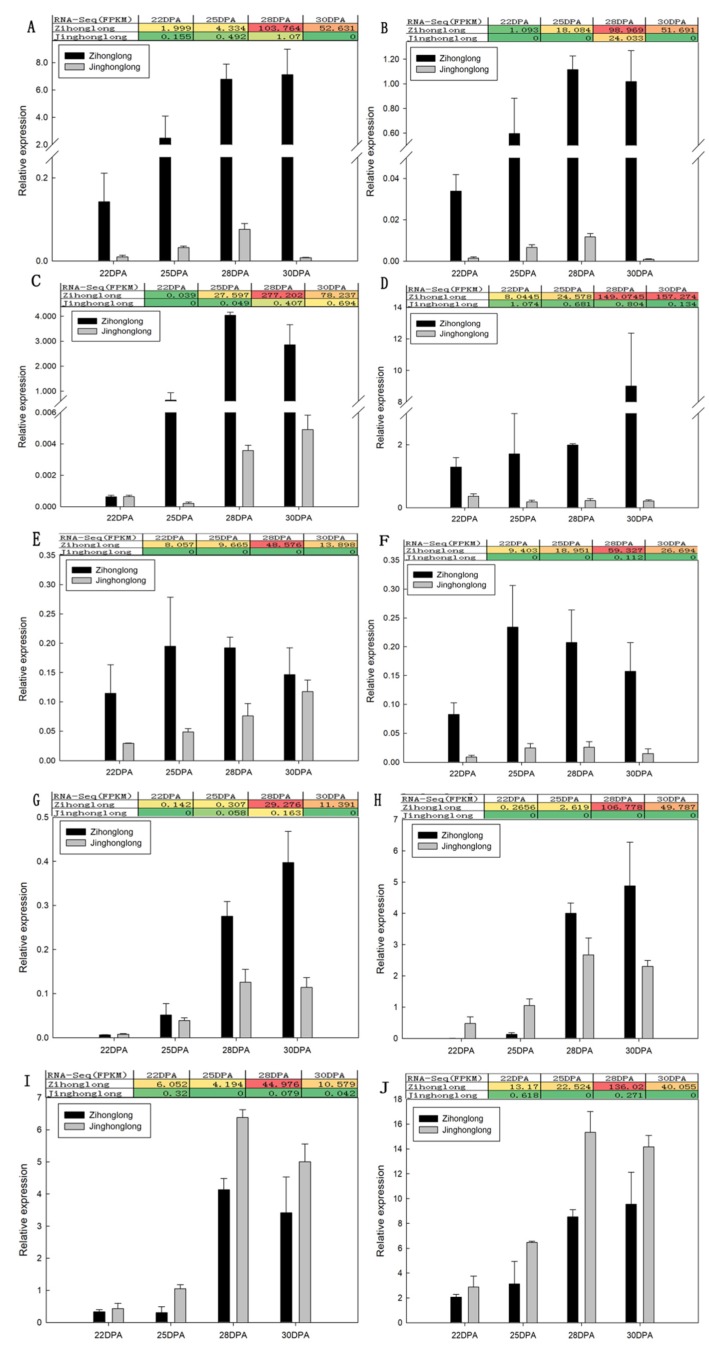
qRT-PCR of candidate gene involved in betalain biosynthesis. (**A**), *HpDOD1*; (**B**), *HpDODA2*; (**C**), *HpCYP76AD4*; (**D**), *HpGSTs*; (**E**), *HpBPE*; (**F**), *HpNAC*; (**G**), *HpCYP704C1*; (**H**), *HpFAR*; (**I**), *HpSTK*; (**J**), *HpVPP1*. Note, the colors in the graph indicate the magnitude of gene expression in the sample. Red indicates that the gene is highly expressed in the sample, yellow indicates that the gene expression is middle, and the blue indicates that the gene expression is low.

To validate the Illumina RNA-Seq transcriptome results, DEGs involved in the biosynthesis of betalain as well as DEGs annotated in Nr or Nt and expressed highly in red pulp but with an FPKM value less than 1 in white pulp were selected from the 173 DEGs as a candidate gene. Subsequently, the 10 DEGs (Table 2) were selected for measurement of transcript levels by qRT-PCR. Furthermore, a comparison between the value of qRT-PCR and that of FPKM was conducted. As illustrated in Figure 5, in general, the quantitative analysis results of the *HpDODA1*, *HpDODA2*, *HpCYP76AD4*, *HpNAC*, *HpGSTs*, and *HpCYP704C1* were consistent with that of the FPKM values. The expression level of *HpDODA1*, *HpDODA2*, *HpCYP76AD4*, *HpNAC*, *HpGSTs*, and *HpCYP704C1* in the pulp of ‘Zihonglong’ increased dramatically at the coloring stage. The qRT-PCR value of *HpDODA1* in ‘Zihonglong’ pulp was 14.16 to 936.34 times than that in ‘Jinghonglong’, and that of *HpDODA2*, *HpCYP704C1*, and *HpGSTs* were a 23.95 to 1146.59, 0.80 to 3.48-fold change, and 3.56 to 42.74 times than in ‘Jinghonglong’, respectively. Moreover, the amount of change in the 4 genes increased with the development of the fruit. The qRT-PCR values of *HpBPE* in ‘Zihonglong’ pulp were higher than that in ‘Jinghonglong’, and the changes were 4.00 to 1.25-fold. The qRT-PCR value of *HpBPE* in ‘Jinghonglong’ pulp exhibited a significant increase tendency with the development of the fruit, while the FPKM values of all four developmental stages were zero. For *HpCYP76AD4*, the qRT-PCR value in ‘Zihonglong’ was 0.99 to 2997.21 times than that in ‘Jinghonglong’ and the maximal value change appeared at 25 DPA, at the pulp color-broken stage. The qRT-PCR value of *HpNAC* in ‘Zihonglong’ pulp was 7.92 to 10.52 times that of ‘Jinghonglong’. Compared to ‘Jinghonglong’, the qRT-PCR value of *HpFAR* in the pulp of ‘Zihonglong’ was 0.01 to 0.13 times during 22 to 25 DPA. However, this increased significantly, and became higher than that in ‘Jinghonglong’ pulp from 28 to 30 DPA; the value was 1.50–2.12 times. The qRT-PCR values of *HpSTK* and *HpVPP1* in pulp of ‘Zihonglong’ were lower than that of ‘Jinghonglong’ at the four development stages; the former was 0.29 to 0.77 and the latter was 0.48 to 0.72 times, respectively, which was inconsistent with the variation of FPKM value.

## 3. Discussion

Fruit is a major source of plant-derived pigments, and the formation of pigment is closely related to the process of fruit development. The L* values decreased and the a*, b* values increased with apple fruit development. A high anthocyanin content may lead to a decrease in fruit brightness [29]. In the present work, the appearance of pigments in ‘Zihonglong’ pulps and the variation characteristic of color parameter was consistent with that of apple [29]. Notably, the color parameters varied predominantly at 25 DPA in the red pup cultivar while the fruit pulp was in the color initiation stage, suggesting that the stage was a crucial period for the accumulation of red pigment.

The proportion of full-length transcripts from Illumina RNA-Seq assembly is very small, and inaccuracy in gene structure characterization resulting from mis-assembly is a common problem, which is exacerbated in the species without a reference genome sequence for the prediction of gene models [30]. Recently, SMRT sequel as a new third-generation sequencing (TGS) platform was carried out by PacBio sequencing. Non-assembled long-read transcripts with low error rate (10%) can be generated by SMRT sequel, and the error rate can be overcome by correction of Illumina RNA-Seq [31]. For example, the mapping rate of long reads in maize can be increased from 11.6 to 99.1% after correction with Illumina read [22]. However, thus far, there has been no report of reference genome sequence or SMRT sequence on pitaya. In the present case, 65,312 and 91,638 genes (non-redundant reads) were generated by SMRT from pooled-stage pulp and corrected by Illumina RNA-Seq. The mean length of the SMRT gene is much longer than that of unigene assembled from Illumina RNA-Seq reads. The pitaya genes had the highest number of hits to the *B. vulgaris* (50.63%), and the species distribution with the greatest number of *H. polyrhizus* was *Vitis vinifera* (50.1%) by Illumina RNA-Seq [6]. Both pitaya and *B. vulgaris* belong to the Caryophyllales order. Therefore, the result illustrates that SMRT data are of higher quality than data from Illumina RNA-Seq.

Transcriptome reconstruction and annotation has been improved significantly with the development of sequencing techniques [30]. Long-read sequencing can provide an efficient reference sequence for plants without a reference genome [17]. Different transcription isoforms in pitaya pulp were detected without a reference genome. A total of 17.42% and 16.97% of genes in the red and white pulp were identified, respectively, including more than 10 isoforms in red pulp (516, 0.79%) in comparison with that of the white pulp (767, 0.84%). SSR markers were considered as an efficient approach to identify genetic diversity in pitaya germplasms and were employed to determine the genetic relationships among pitaya species [32]. The identified SSRs from the SMRT data can facilitate the identification of genetic diversity in pitaya.

LncRNAs are key regulatory molecules that regulate gene expression and have become a hot topic in biology [33,34]; 11,046 lncRNAs were predicted in *Salvia miltiorrhiza* [21]. In maize, 867 transcripts with a mean length of 1.1 kb were identified as novel high-confidence lncRNAs [22]. A total of 417 and 531 lncRNAs were identified in sweet potato and *I. trifida*, respectively [33], 223 and 205 lncRNAs were obtained in the leaf and root of *Astragalus membranaceus*, respectively [30], 2426 transcript sequences including 1220 non-ORF transcript sequences candidate lncRNAs were identified in sugarcane [25]. In the present work, 11,650 and 11,113 lncRNAs were identified with four analytical methods informing the red and white pulp, respectively, which is more than from other documented species. However, their functions require further investigations.

Compared with many other analysis methods, WGCNA has the advantages of summarizing and standardizing the methods and functions of integrated R packages [35]. Moreover, combining the WGCNA method and RNA-Seq data can be used to better mine the genes and transcription factors related to the traits [36]. In this study, the WGCNA method was used for the first time in pitaya fruit, and the modules related to pigmentation traits were identified. Betelain, an important pigment in most Caryophyllales plants, can be used as a natural colorant in food [37], cosmetics, and pharmaceuticals [38]. Intensive attempts have been focused on the betalain biosynthesis and genes function, and much more betalain-related candidate genes, such as TYR [39,40], *BvMYB1* [15], CYP76AD1 [10], and *BvDODA1* [13] were identified. Even so, research regarding betalain-related genes, especially for pitaya, has thus far been limited.

Betalamic acid is the chromophore molecule of both betacyanins and betaxanthins, and cDOPA as well as its derivatives are essential to produce betacyanin [41]. The formation of betalamic acid and cDOPA are crucial in betacyanin synthesis; the absence of betalamic acid may block the production of betalain. In white pulp cultivar, neither red betacyanins nor yellow betaxanthins were detected in the pulp. Hence, we hypothesized that CYP76AD and DODA were crucial genes to the formation of betalain. The expression level of *HpCYP76AD4*, *HpDODA1*, and *HpDODA2* were remarkably higher in the red pulp than that in the white pulp. Meanwhile, another cytochrome p450, *HpCYP704C1* was identified, and both the expression level and change variation between the two samples was less than that of *HpCYP76AD4*. At all of the four development stages, the expression level in the pulp of ‘Zihonglong’ was significantly higher than that of ‘Jinghonglong’. Therefore, the three genes may facilitate the biosynthesis of betalain in pitaya pulp.

*BvMYB1* is currently the only known betalain-related transcription factor, which has an essential role as a positive regulator of betalain biosynthesis through activation of the CYP76AD1 and *BvDODA1* genes [7]. In apple, two NAC TF of *MdNAC029* and *MdNAC52* were confirmed to be participating in anthocyanin biosynthesis. *MdNAC029* may positively regulate anthocyanin accumulation by directly promoting the expression of *MdMYB1* gene [42]. *MdNAC52* binds to the promoters of *MdMYB9* and *MdMYB11* to promote anthocyanin and PA biosynthesis, and directly regulates LAR to modulate PA metabolism [43]. *MdGSTF6* was an anthocyanin transporter, and the knockdown of *MdGSTF6* by RNA interference inhibited anthocyanin accumulation in apple seedlings [44]. These TF and genes involved in the biosynthesis of anthocyanin were verified in apple. The expression level of *HpNAC* and *HpGSTs* were consistent with the variation of color in pitaya pulp; therefore, they may participate in the biosynthesis of betalain in pitaya pulp.

**Figure 6:**
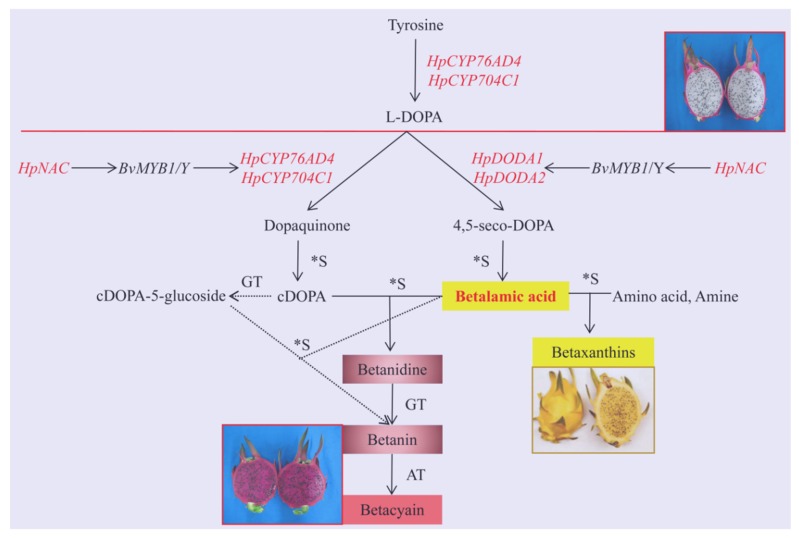
The putative betalain biosynthesis pathway from pitaya fruit. Note, the genes identified in red words were derived from our pitaya pulp data. The solid line arrows represent the common biosynthetic pathway of betalain, and the dotted line arrows designate reactions of an alternative pathway.

In summary, full-length transcripts of pitaya pulp that are generated from SMRT with Illumina RNA-seq provide an efficient process to the research of genes and facilitate the identification of additional betalain-related genes. *HpCYP76AD4*, *HpDODA1*, *HpDODA2*, and *HpCYP76AD4* involved in betalain biosynthesis were identified in pitaya fruit, and *HpNAC* and *HpGSTs* might participate in the regulation of betalain in pitaya fruit. Furthermore, *HpNAC* may play a role in the regulation of betalain synthesis in coordination with MYB TF (Figure 6), which provides new insights into betalain synthesis in pitaya.

## 4. Material and Methods

### 4.1. Plant Materials

The seedlings of *H. polyrhizus* cv. Zihonghlong and *H. undatus* cv. Jinghonglong, cultivated in Langdang fruit professional cooperative (Luodian, Guizhou province, China.), were used in this study. All plants were planted in 2009. Three hundred flowers blooming on the same day were marked with tags in 2016, and thirty labelled healthy fruits of each four developmental stages from 29th June to 7th July (22nd, 25th, 28th, and 30th after anthesis) were collected randomly from different plants (Figure 1A). All samples intended for RNA extraction were fresh-frozen in liquid nitrogen immediately after collection and stored at −80 °C until use.

### 4.2. Measurements of Color and Betalain

For color analyses, L*, a*, and b* of pitaya pulp were measured with CR-10 Chromaportable colorimeter (Konica Minolta Sensing, Inc., Osaka, Japan). All determinations were performed in duplicate. L* value represented the relative lightness of colors ranging from 0 (black) to 100 (white). Values of a* and b* ranged from −60 to 60, where a* was negative for green color and positive for red color, and b* was negative for blue and positive for yellow [29,45]. The concentration of betacyanin and betaxanthin was detected according to Wu et al. [46].

### 4.3. RNA Sample Preparation

Total RNA was isolated using the RNeasy Plus Mini Kit (Qiagen, Valencia, CA, USA), respectively. The purity and concentration of RNA were measured using the NanoDrop ND-1000 spectrophotometer (NanoDrop Technologies, Rockland, DE, USA) with an OD260/280 reading. The integrity of the RNA was determined on agarose gel electrophoresis with the Agilent 2100 Bioanalyzer (Agilent Technologies, CA, USA).

### 4.4. Library Preparation and SMRT Sequencing

The libraries were produced and sequenced by Shanxi Breeding Biotechnologies Technology Co., Ltd. First, mRNA was enriched by Oligo (dT) magnetic beads, then the enriched mRNA was reverse transcribed into full length 1st strand cDND using Clontech SMARTer PCR cDNA Synthesis Kit. PCR cycle optimization was used to determine the optimal amplification cycle number for the downstream large-scale application. The optimized cycle number was used to generate double-stranded cDNA, followed optional size (>4 kb) selection using the BluePippinTM for combined SMRT bell library. Full length cDNAs were performed, DNA damage repaired, end repaired, and ligated to sequencing adapters, and then digested with exonuclease. Qualified libraries were sequenced on the PacBio Sequel (Pacific Bio-science Inc., CA, USA) platform according to the effective concentration and data output requirements of the library.

### 4.5. Preprocessing of SMRT Reads

The subreads were acquired from raw sequencing reads using the SMRT Link v5.0 (minLength = 200, minReadScore = 0.75) pipeline supported by Pacific Biosciences, and CCS reads were extracted out of subreads’ BAM file. Through RS_IsoSeq (minPasses = 1, minPredicted Accuracy = 0.8), CCS reads were classified into full-length non-chimeric (FLNC), non-full-length (NFL) based on cDNA primers and polyA tail signal. Subsequently, the FLNC reads were clustered by Iterative Clustering for Error Correction (ICE) software to generate the cluster consensus isoforms [47]. Then, NFL reads were used to polish the above obtained cluster consensus isoforms by Quiver (www.pacbiodevnet.com/Quiver) to finally obtain the FLNC high quality polished consensus sequences (accuracy ≥99%). After being corrected by SGS using LoRDEC, non-redundant high-quality full-length transcripts were generated by CD-HIT (c = 0.99) for further analysis [48].

### 4.6. Functional Annotation of Genes

Non-redundant transcript sequence as genes obtained after CD-HIT deduplication were grouped and mapped to nine protein and nucleic acid database to obtain the annotation information of the gene. These databases included NR, Nt, Swissprot [49], GO [50], COG [51], KOG, Pfam [52], TrEMBL [49], and KEGG [53]. GO annotation was analyzed by Blast2GO software with Nr annotation results of genes. Genes ranking the first 20 highest score and no shorter than 33 HSPs (High-Scoring Segment Pair) hits were selected to conduct Blast2GO analysis. Then, functional classification of genes was run using WEGO software.

### 4.7. SSR Detection

The MicroSAtellite identification tool (MISA; http://pgrc.ipk-gatersleben. de/misa/) was employed for microsatellite mining in the whole transcriptome. Mononucleotide, dinucleotide, trinucleotide, tetranucleotide, pentanucleotide, hexanucleotide, and compound SSR were identified by analyzing transcript sequences.

### 4.8. lncRNAs Prediction

The coding potential of transcripts were predicted by predictor of long non-coding RNAs and messenger RNAs based on an improved k-mer scheme (PLEK) [54] and Coding-Non-Coding Index (CNCI) [55]. Then, transcriptional sequences predicted from PLEK and CNCI were blasted with the known protein database using Coding Potential Calculator (CPC) [56]. The transcriptional sequences predicted by PLEK, CNCI, and CPC software underwent hmmscan homologous search with Pfam [52] database, and finally the LncRNA sequences were obtained.

### 4.9. Next Generation Sequencing

Total RNA (5μg) was digested by using DNase I (NEB, Frankfurt, Germany). The sample was purified with Agencourt RNAClean XP Beads and fragmented into 130–170 nt. First-strand cDNA was generated by First Strand Master Mix and Super Script II reverse transcription (Invitrogen). Then second-strand cDNA was synthesized using Second Strand Master Mix. After end repairing, adding A and adaptor ligation, several rounds of PCR amplification with PCR Primer Cocktail and PCR MasterMix were performed to enrich the cDNA fragments. The final library is quantitated by using the Agilent 2100 bioanalyzer instrument. The qualified libraries were sequenced pair-end on the Illumina HiSeq 4000 System.

### 4.10. Identification of DEGs Involved in Betalain Biosysthensis

Data from Illumina RNA-Seq were mapped to the non-redundant SMRT reference by RSEM software. The expression abundance of unigene was represented as value of FPKM, and differential expression gene (FDR <0.01 and FC ≥ 2) were obtained using EBSeq [57]. Genes with more than one FPKM value above 10 were selected. Combining the value of a* as well as the content of betacyanin, betaxanthin, betalain (calculated as the sum of betacyanin and betaxanthin), and the FPKM value of the gene, a WGCNA was performed using an R package to identify the module associated with betalain. Quantitative real-time PCR (qRT-PCR) analysis was conducted on ten genes in the WGCNA module involved in betalain or associated with color qRT-PCR analysis was performed according to Nie et al. [58]. Actin YLS8 (GenBank ID 356278) was used as a reference gene. Specific primers were designed using primer 5 (Table S4). Data was analyzed using 2^−ΔΔ*C*T^ method.

### 4.11. Data Processing and Analysis

Color parameter and betalain content data were processed with Excel (Microsoft, Washington, USA); then, one-way ANOVA was employed for statistical analysis of color parameter and betalain concentrations, as well as for significant analysis of color parameters, followed by a Duncan’s multiple range test at the 5% level (*p* ≤ 0.05) in SPSS17.0 (SPSS Inc., Chicago, IL, USA). 

## Figures and Tables

**Table 1:** Pitaya long-read sequencing transcriptome annotation with different databases.

Annotated-Database	Annotated-Number	Percentage (%)
COG-Annotation	34,601	28.69
GO-Annotation	54,706	45.36
KEGG-Annotation	28,796	23.88
KOG-Annotation	56,010	46.44
Pfam-Annotation	88,549	73.42
Swissprot-Annotation	72,130	59.81
TrEMBL-Annotation	95,458	79.15
nr-Annotation	105,413	87.40
nt-Annotation	63,052	52.28
All-Annotation	120,604	100.00

**Table 2:** The list of 10 DEGs used for qRT-PCR.

Gene	Gene ID	Gene	Gene ID
*HpDODA1*	i1_LQ_R_c96099/f1p0/1004	*HpNAC*	i1_HQ_R_c77544/f11p0/1295
*HpDODA2*	i1_HQ_R_c9184/f4p0/1375	*HpCYP704C1*	i1_LQ_R_c24611/f1p0/1636
*HpCYP76AD4*	i1_HQ_R_c13003/f5p0/1979	*HpFAR*	i1_HQ_R_c76874/f3p0/1664
*HpGSTs*	i1_LQ_R_c13451/f1p0/1160	*HpSTK*	i2_HQ_R_c697/f6p0/2233
*HpBPE*	i1_LQ_R_c9617/f1p0/1492	*HpVPP1*	i2_HQ_R_c679/f4p0/2556

## Supplementary Materials

Supplementary materials can be found at https://www.mdpi.com/1422-0067/21/9/3288/s1.
