# Supplementary material for: Comparative Transcriptome Analysis Combining SMRT- and Illumina-Based RNA-Seq Identifies Potential Candidate Genes Involved in Betalain Biosynthesis in Pitaya Fruit
Source: Int J Mol Sci. 2020 May 6;21(9):3288. doi: 10.3390/ijms21093288 (PMC7246777; doi:10.3390/ijms21093288)
Supplement: Supplementary file 1 [file ijms-21-03288-s001.zip › Supplementary materials/Table S1.docx]

[Supplementary](javascript:;) table 1

Comparison of SMRT sequencing between ‘Zihonglong’ and ‘Jinghonglong’

| Data | | ‘Zihonglong’ | ‘Jinghonglong’ |
| --- | --- | --- | --- |
| Subreads | base(G) | 8.47 | 7.74 |
|  | Number | 9,579,839 | 7,245,659 |
| CCS reads | Total | 367,001 | 481,602 |
|  | FL | 322,995 | 366,583 |
|  | FLNC | 314,173 | 348,184 |
|  | NFL | 43,599 | 114,621 |
| Polished consensus sequences | Total | 184,875 | 188,215 |
|  | High quality | 23,669 | 25,299 |
|  | Low quality | 161,206 | 162,916 |
| Corrected consensus | | 184,875 | 188,215 |
| Genes | | 65,317 | 91,638 |
