# Supplementary material for: Comparative Transcriptome Analysis Combining SMRT- and Illumina-Based RNA-Seq Identifies Potential Candidate Genes Involved in Betalain Biosynthesis in Pitaya Fruit
Source: Int J Mol Sci. 2020 May 6;21(9):3288. doi: 10.3390/ijms21093288 (PMC7246777; doi:10.3390/ijms21093288)
Supplement: Supplementary file 1 [file ijms-21-03288-s001.zip › Supplementary materials/Table S2.docx]

[Supplementary](javascript:;) table 2

Comparison results between SMRT sequencing transcript and Illumina sequencing unigene.

| Length Distribution (nt) | SMRT gene number | | Illumina sequencing assembled unigene number | |
| --- | --- | --- | --- | --- |
|  | ‘Zihonglong’ | ‘Jinghonglong’ | ‘Zihonglong’ | ‘Jinghonglong’ |
| 200-300 | 503 | 265 | 43,646 | 38,796 |
| 300-500 | 7,497 | 5,081 | 26,714 | 24,097 |
| 500-1000 | 29,237 | 33,559 | 18,441 | 17,436 |
| 1000-2000 | 21,462 | 40,471 | 10,459 | 10,174 |
| 2000-3000 | 4,700 | 9,579 | 4,714 | 4,424 |
| 3000+ | 1,918 | 2,683 | 3,187 | 2,984 |
| Total Number | 65,317 | 91,638 | 107,161 | 97,911 |
| Total Length | 76,752,116 | 122,496,823 | 72,942,534 | 68,104,067 |
| N 50 Length | 975 | 1,385 | 1,169 | 1,208 |
| Mean length | 1,175 | 1,337 | 681 | 696 |
