# Supplementary material for: Comparative Transcriptome Analysis Combining SMRT- and Illumina-Based RNA-Seq Identifies Potential Candidate Genes Involved in Betalain Biosynthesis in Pitaya Fruit
Source: Int J Mol Sci. 2020 May 6;21(9):3288. doi: 10.3390/ijms21093288 (PMC7246777; doi:10.3390/ijms21093288)
Supplement: Supplementary file 1 [file ijms-21-03288-s001.zip › Supplementary materials/Table S3.docx]

[Supplementary](javascript:;) table 3

The gene ID list selected from WGCNA

| **Num** | **Gene ID** | | **Num** | **Gene ID** |
| --- | --- | --- | --- | --- |
| 1 | i0_HQ_R_c1639/f24p4/610 | | 88 | i1_HQ_R_c88047/f37p0/1049 |
| 2 | i0_HQ_R_c1847/f22p7/977 | | 89 | i1_HQ_R_c88138/f114p0/1460 |
| 3 | i0_HQ_R_c208282/f3p22/987 | | 90 | i1_HQ_R_c9184/f4p0/1375 |
| 4 | i0_HQ_R_c208834/f7p0/497 | | 91 | i1_HQ_W_c8817/f2p0/1805 |
| 5 | i0_HQ_R_c209282/f5p0/979 | | 92 | i1_LQ_R_c13451/f1p0/1160 |
| 6 | i0_HQ_R_c211275/f5p3/640 | | 93 | i1_LQ_R_c14192/f1p0/1935 |
| 7 | i0_HQ_R_c271714/f21p6/620 | | 94 | i1_LQ_R_c15780/f1p0/1045 |
| 8 | i0_HQ_R_c296799/f2p6/646 | | 95 | i1_LQ_R_c20481/f1p0/1976 |
| 9 | i0_HQ_R_c29987/f4p2/996 | | 96 | i1_LQ_R_c21659/f1p0/1995 |
| 10 | i0_HQ_R_c33433/f5p24/778 | | 97 | i1_LQ_R_c22025/f1p0/1093 |
| 11 | i0_HQ_R_c342955/f5p3/722 | | 98 | i1_LQ_R_c22459/f1p0/1841 |
| 12 | i0_HQ_R_c350883/f2p5/737 | | 99 | i1_LQ_R_c22888/f1p0/1622 |
| 13 | i0_HQ_R_c418743/f7p12/766 | | 100 | i1_LQ_R_c22909/f1p0/1411 |
| 14 | i0_HQ_R_c419723/f11p5/816 | | 101 | i1_LQ_R_c24611/f1p0/1636 |
| 15 | i0_HQ_R_c44093/f10p2/790 | | 102 | i1_LQ_R_c26279/f1p1/1258 |
| 16 | i0_HQ_R_c48608/f3p0/671 | | 103 | i1_LQ_R_c26409/f1p0/1507 |
| 17 | i0_HQ_R_c507545/f6p17/815 | | 104 | i1_LQ_R_c26523/f1p0/1248 |
| 18 | i0_HQ_R_c602818/f48p5/697 | | 105 | i1_LQ_R_c29284/f1p0/1187 |
| 19 | i0_HQ_R_c603520/f33p14/749 | | 106 | i1_LQ_R_c31367/f1p0/1730 |
| 20 | i0_HQ_R_c604376/f3p10/813 | | 107 | i1_LQ_R_c31763/f1p0/1052 |
| 21 | i0_HQ_R_c689370/f4p0/798 | | 108 | i1_LQ_R_c43546/f1p0/1094 |
| 22 | i0_HQ_R_c689469/f5p3/785 | | 109 | i1_LQ_R_c43986/f1p0/1985 |
| 23 | i0_HQ_R_c8843/f6p2/962 | | 110 | i1_LQ_R_c46047/f1p0/1169 |
| 24 | i0_HQ_W_c141818/f2p23/827 | | 111 | i1_LQ_R_c58553/f1p0/1772 |
| 25 | i0_HQ_W_c327549/f9p27/788 | | 112 | i1_LQ_R_c58615/f1p4/1336 |
| 26 | i0_HQ_W_c55132/f11p2/657 | | 113 | i1_LQ_R_c59116/f1p0/1235 |
| 27 | i0_LQ_R_c11478/f1p0/962 | | 114 | i1_LQ_R_c59839/f1p0/1338 |
| 28 | i0_LQ_R_c125588/f1p2/597 | | 115 | i1_LQ_R_c60477/f1p0/1209 |
| 29 | i0_LQ_R_c127116/f1p2/514 | | 116 | i1_LQ_R_c60931/f1p0/1370 |
| 30 | i0_LQ_R_c13166/f1p405/654 | | 117 | i1_LQ_R_c61865/f1p0/1997 |
| 31 | i0_LQ_R_c163939/f1p4/786 | | 118 | i1_LQ_R_c65030/f1p0/1385 |
| 32 | i0_LQ_R_c176946/f1p2/545 | | 119 | i1_LQ_R_c65784/f1p0/1615 |
| 33 | i0_LQ_R_c209768/f5p1/981 | | 120 | i1_LQ_R_c66470/f1p0/1915 |
| 34 | i0_LQ_R_c230547/f1p4/568 | | 121 | i1_LQ_R_c67714/f1p0/1697 |
| 35 | i0_LQ_R_c287188/f2p5/512 | | 122 | i1_LQ_R_c72552/f1p0/1090 |
| 36 | i0_LQ_R_c296838/f1p0/692 | | 123 | i1_LQ_R_c74252/f1p0/1020 |
| 37 | i0_LQ_R_c307769/f1p2/670 | | 124 | i1_LQ_R_c87848/f12p0/1464 |
| 38 | i0_LQ_R_c345859/f1p4/786 | | 125 | i1_LQ_R_c89711/f1p0/1062 |
| 39 | i0_LQ_R_c364555/f1p1/872 | | 126 | i1_LQ_R_c96099/f1p0/1004 |
| 40 | i0_LQ_R_c391001/f1p2/418 | | 127 | i1_LQ_R_c9617/f1p0/1492 |
| 41 | i0_LQ_R_c438713/f2p4/885 | | 128 | i1_LQ_W_c61059/f1p37/1500 |
| 42 | i0_LQ_R_c507434/f10p9/895 | | 129 | i1_LQ_W_c64798/f1p0/1189 |
| 43 | i0_LQ_R_c51426/f1p3/598 | | 130 | i1_LQ_W_c76763/f1p0/1518 |
| 44 | i0_LQ_R_c524122/f1p0/492 | | 131 | i2_HQ_R_c1032/f3p0/2177 |
| 45 | i0_LQ_R_c538795/f1p1/593 | | 132 | i2_HQ_R_c1124/f2p0/2896 |
| 46 | i0_LQ_R_c543039/f1p6/1001 | | 133 | i2_HQ_R_c263/f2p0/2759 |
| 47 | i0_LQ_R_c56450/f1p497/905 | | 134 | i2_HQ_R_c599/f7p0/2394 |
| 48 | i0_LQ_R_c58307/f1p14/565 | | 135 | i2_HQ_R_c679/f4p0/2556 |
| 49 | i0_LQ_R_c58783/f1p40/774 | | 136 | i2_HQ_R_c697/f6p0/2233 |
| 50 | i0_LQ_R_c604271/f3p0/665 | | 137 | i2_HQ_R_c8225/f22p0/2122 |
| 51 | i0_LQ_R_c61346/f1p6/1031 | | 138 | i2_HQ_R_c8460/f10p0/2063 |
| 52 | i0_LQ_R_c6253/f1p0/895 | | 139 | i2_HQ_R_c8461/f26p0/2202 |
| 53 | i0_LQ_R_c648137/f1p9/520 | | 140 | i2_HQ_R_c923/f3p7/2153 |
| 54 | i0_LQ_R_c709499/f1p0/761 | | 141 | i2_HQ_W_c1144/f5p0/2685 |
| 55 | i0_LQ_R_c711362/f1p4/330 | | 142 | i2_HQ_W_c931/f6p0/2369 |
| 56 | i0_LQ_R_c78992/f1p2/612 | | 143 | i2_LQ_R_c1765/f1p0/2777 |
| 57 | i0_LQ_R_c79410/f1p7/845 | | 144 | i2_LQ_R_c2436/f1p1/2121 |
| 58 | i0_LQ_R_c8474/f1p12/683 | | 145 | i2_LQ_R_c2851/f1p0/2093 |
| 59 | i0_LQ_W_c243004/f1p14/744 | | 146 | i2_LQ_R_c3475/f1p3/2151 |
| 60 | i0_LQ_W_c26437/f2p1/632 | | 147 | i2_LQ_R_c4250/f1p0/2092 |
| 61 | i0_LQ_W_c346252/f1p190/592 | | 148 | i2_LQ_R_c5085/f1p0/2376 |
| 62 | i1_HQ_R_c11045/f2p1/1437 | | 149 | i2_LQ_R_c6530/f1p0/2154 |
| 63 | i1_HQ_R_c1298/f8p0/1759 | | 150 | i2_LQ_R_c6804/f1p5/2602 |
| 64 | i1_HQ_R_c16489/f2p0/1048 | | 151 | i2_LQ_R_c6861/f1p0/2281 |
| 65 | i1_HQ_R_c1840/f11p0/1733 | | 152 | i2_LQ_R_c7446/f1p0/2419 |
| 66 | i1_HQ_R_c21833/f9p0/1249 | | 153 | i2_LQ_R_c7873/f1p0/2042 |
| 67 | i1_HQ_R_c2242/f5p0/1194 | | 154 | i2_LQ_R_c8585/f1p1/2290 |
| 68 | i1_HQ_R_c26542/f2p0/1374 | | 155 | i2_LQ_W_c14355/f1p0/2019 |
| 69 | i1_HQ_R_c27300/f2p0/1385 | | 156 | i2_LQ_W_c6900/f1p0/2654 |
| 70 | i1_HQ_R_c30995/f2p0/1037 | | 157 | i2_LQ_W_c7141/f1p0/2590 |
| 71 | i1_HQ_R_c33371/f25p0/1328 | | 158 | i3_HQ_R_c273/f2p0/3469 |
| 72 | i1_HQ_R_c33632/f3p0/1296 | | 159 | i3_LQ_R_c407/f1p0/3783 |
| 73 | i1_HQ_R_c33807/f10p0/1125 | | 160 | i3_LQ_R_c686/f1p0/4138 |
| 74 | i1_HQ_R_c36615/f3p0/1990 | | 161 | i3_LQ_R_c810/f1p0/3572 |
| 75 | i1_HQ_R_c36832/f2p0/1870 | | 162 | i3_LQ_R_c850/f1p0/3186 |
| 76 | i1_HQ_R_c36889/f2p0/1244 | | 163 | i4_LQ_R_c255/f1p0/5053 |
| 77 | i1_HQ_R_c53643/f2p0/1053 | | 164 | i4_LQ_R_c298/f1p0/4222 |
| 78 | i1_HQ_R_c53819/f2p0/1640 | | 165 | i4_LQ_R_c314/f1p0/4933 |
| 79 | i1_HQ_R_c57032/f3p0/1222 | | 166 | i4_LQ_R_c84/f1p0/4270 |
| 80 | i1_HQ_R_c57202/f2p7/1502 | | 167 | i5_LQ_R_c73/f1p0/5847 |
| 81 | i1_HQ_R_c76341/f38p0/1381 | | 168 | i6_HQ_R_c551/f254p0/6605 |
| 82 | i1_HQ_R_c76368/f9p0/1170 | | 169 | i6_LQ_R_c331/f1p0/6530 |
| 83 | i1_HQ_R_c76727/f30p0/1467 | | 170 | i6_LQ_R_c458/f1p0/6694 |
| 84 | i1_HQ_R_c76874/f3p0/1664 | | 171 | i6_LQ_R_c511/f1p0/6329 |
| 85 | i1_HQ_R_c77544/f11p0/1295 | | 172 | i7_LQ_R_c20/f1p0/8031 |
| 86 | i1_HQ_R_c86519/f44p0/1453 | | 173 | i9_LQ_R_c10/f1p0/9078 |
| 87 | i1_HQ_R_c86949/f2p0/1073 |  | | |
