# Supplementary material for: Comparative Transcriptome Analysis Combining SMRT- and Illumina-Based RNA-Seq Identifies Potential Candidate Genes Involved in Betalain Biosynthesis in Pitaya Fruit
Source: Int J Mol Sci. 2020 May 6;21(9):3288. doi: 10.3390/ijms21093288 (PMC7246777; doi:10.3390/ijms21093288)
Supplement: Supplementary file 1 [file ijms-21-03288-s001.zip › Supplementary materials/Table S4.docx]

[Supplementary](javascript:;) table 4

The primer sequence list of ten candidate genes

| Number | Gene | Prime |
| --- | --- | --- |
| 1 | >i1_LQ_R_c96099/f1p0/1004 | F:GGGTGTTGGCAAAGAAGTGTC |
|  |  | R:GGAACATCGCTGAAATCGTAAA |
| 2 | >i1_HQ_R_c9184/f4p0/1375 | F:TTACGATTTCAGCGATGTTCCT |
|  |  | R:GCCAAAGCCCTCCCTATGTC |
| 3 | >i1_HQ_R_c13003/f5p0/1979 | F:AATCCGACGATGATGGAGAAAG |
|  |  | R:GGAGGGTGTAGACGCAATGTTT |
| 4 | >i1_HQ_R_c76874/f3p0/1664 | F:TATGGGCGACACTCTAAATGGA |
|  |  | R:GGTGACGATAGTAGGGCGAAC |
| 5 | >i1_HQ_R_c77544/f11p0/1295 | F:CAAATCAAGGCAAGAAATCCC |
|  |  | R:CCAAAGTCTGTCTTCTCGGGTAA |
| 6 | >i1_LQ_R_c13451/f1p0/1160 | F:AGAGCAGGAGGCAGCAAAGA |
|  |  | R:ACATCAACGAACCCAAACGAG |
| 7 | >i1_LQ_R_c24611/f1p0/1636 | F:GAGCAAGAAAGACCCAGAGCA |
|  |  | R:GAGGCAACAGATAAACCAAGTGAT |
| 8 | >i1_LQ_R_c9617/f1p0/1492 | F:GCCACAGCCTCGCAGAAA |
|  |  | R:AGTGCCTTGCCAATGACCTTA |
| 9 | >i2_HQ_R_c679/f4p0/2556 | F:CAATTCAACACCATCCCAGGA |
|  |  | R:CAGCGAGAACGCCAGAAAGA |
| 10 | >i2_HQ_R_c697/f6p0/2233 | F:AGGTGCTTGGGATGGAGGT |
|  |  | R:TATCCAGGCGTTGTAGAATCGT |
