# Supplementary figures and images for: Comparative Transcriptome Analysis Combining SMRT- and Illumina-Based RNA-Seq Identifies Potential Candidate Genes Involved in Betalain Biosynthesis in Pitaya Fruit
Source: Int J Mol Sci. 2020 May 6;21(9):3288. doi: 10.3390/ijms21093288 (PMC7246777; doi:10.3390/ijms21093288)

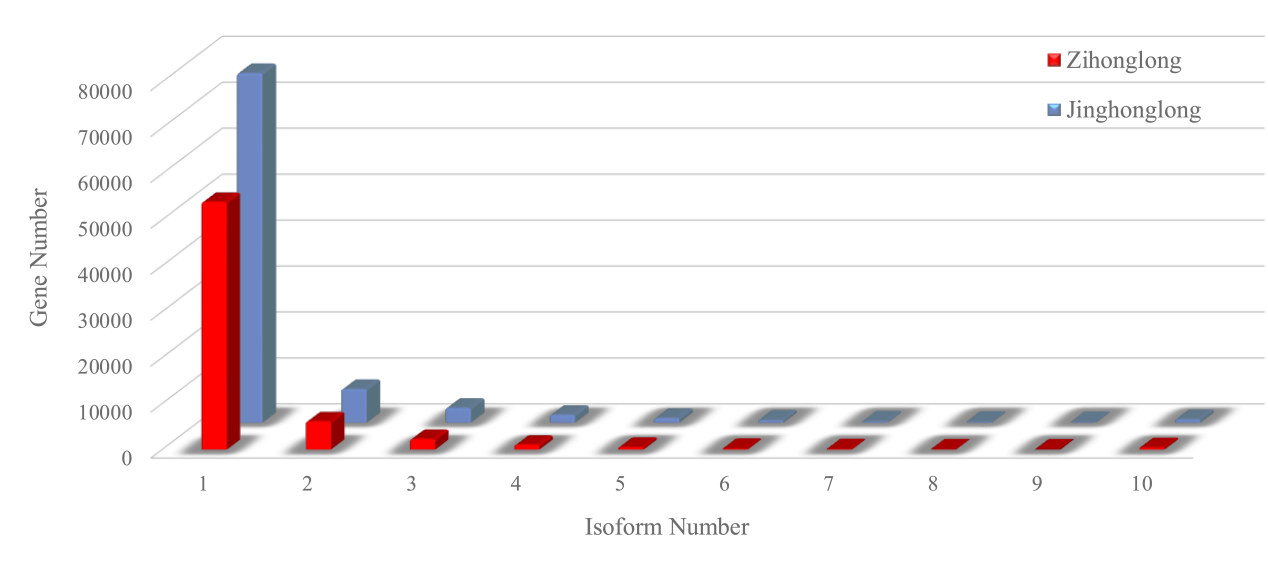


Figure S1. Isoform number per gene（X-axis: isoform number；Y-axis: gene number）

Supplement: Supplementary file 1 [file ijms-21-03288-s001.zip › Supplementary materials/Figure S1 containg caption.docx]

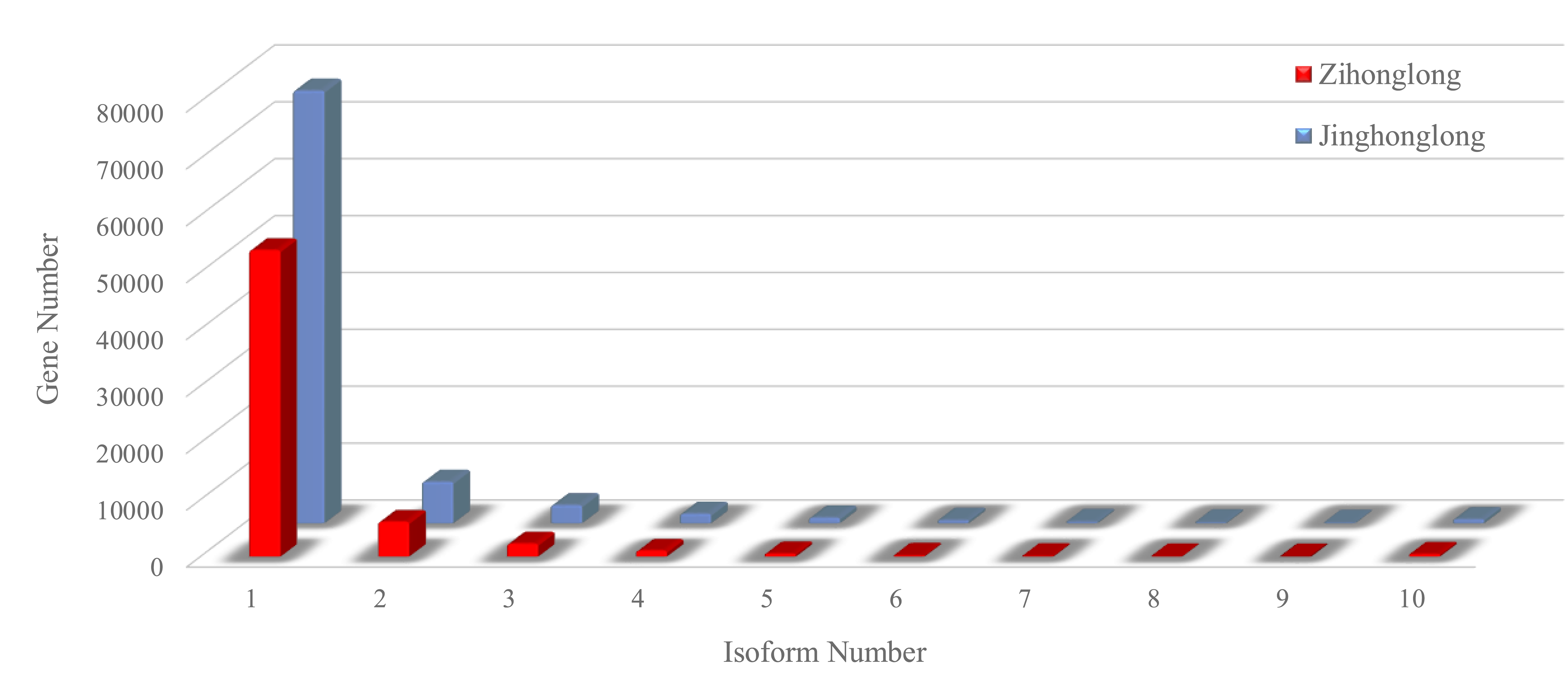

Supplement: Supplementary file 1 [file ijms-21-03288-s001.zip › Supplementary materials/Figure S1. Isoform number per gene.tif]
